# Medical Items

**Published:** 1895-04

**Authors:** 


### *MEDICAL ITEMS.*

THE American Medical Association now numbers 3,920 members

PREMIUMS.—If our cash-in-advance subscribers prefer they can get the book, Antisepsis and Antiseptics, with THE JOURNAL instead of a thermometer. The book is described on page IX.

DR. DUJARDIN BEAUMETZ, the distinguished authority in physiology and therapeutics, died in Paris, February 16th. For several years he had been editor-in-chief of the *Bulletin General de Therapeutique.*

THE persons who drum for doctors at Hot Springs, Ark., are now required to wear a metallic plate on the left lapel of the outside coat, on which plate must be inscribed the name of the employing doctor.—*Ex.*

OUR thanks are due Secretary R. D. Jewett, of Wilmington, for the transactions of the forty-first annual meeting of the North Carolina Medical Society. The volume contains a number of interesting and well-written papers. The transactions deserve a better binding.

So far as figures are reliable the mortality from cancer seems to be on the increase in the United States. For every 100,000 of the population, in 1850, there were nine deaths from cancer; in 1860, eleven deaths; in 1870, sixteen; in 1880, twenty-six, and in 1890, thirty-three deaths.

A WESTERN doctor advertises thus: "Legs and arms sawed off while you wate without pane. Childbirth and tumors a specialty. Coleck, cramps, costiveness and worms nailed on sight. Wringworms, pole-evil, moles and cross-eye cured in one treatment.

P. S.—Cash in advance. N. B.—No coroner never yet sot on the remanes of my customers, and enny one hiring me doant have to buy a gravestone."

---

ANOTHER claimant arises for the honor of the first American symphyseotomy. Dr. Joel O. Williams, of William Penn, Texas, is said to have done this operation in 1880, 1884, and in 1889. Dr. Robert P. Harris, in the *Medical News*, vouches for the authenticity of these operations. Dr. Williams says that the circumstances under which his first operation was done were such as to prevent his making a prompt report of it. The claim of Dr. Williams seems to rest on better basis than that once raised by Dr. Coggin, of Athens, Ga.

---

THE first meeting of the Regular Examining Board for examining applicants to practice was held April 5th. There were about seventy applicants. All passed. We hope to print the examinations in full in our next issue.

At a late meeting of the Eclectic Examining Board there were eleven applicants for license. All passed.

Speaking of boards, we learn from the daily papers (advt.) that the secretary of the Eclectic Board is a "specialist in all chronic diseases of men, women, and children."

---

A COMMITTEE has been formed for the purpose of investigating the liquor question in its ethical, legislative, and physiological bearings. Among its members are Cardinal Gibbons, Rabbi Gottheil, Archbishop Ireland, Bishop Andrews, Professor Bowditch of Harvard University, Dr. J. S. Billings, Dr. Weir Mitchell, President Elliot, and Cornelius Vanderbilt. From these and other names on the list of members, it is evident that the order of talent engaged in this work is such that great results are to be expected. Physiological experiments on an extensive scale will be made, and nothing spared to make the investigation one of the most exhaustive character.—*Memphis Medical Monthly.*

